# Resident Innate Immune Cells in the Cornea

**DOI:** 10.3389/fimmu.2021.620284

**Published:** 2021-02-26

**Authors:** Jun Liu, Zhijie Li

**Affiliations:** International Ocular Surface Research Center, Institute of Ophthalmology, and Key Laboratory for Regenerative Medicine, Jinan University Medical School, Guangzhou, China

**Keywords:** cornea, immune cells, macrophages, mast cells, Langerhans cells, innate lymphoid cells, γδ T-cells

## Abstract

The cornea is a special interface between the internal ocular tissue and the external environment that provides a powerful chemical, physical, and biological barrier against the invasion of harmful substances and pathogenic microbes. This protective effect is determined by the unique anatomical structure and cellular composition of the cornea, especially its locally resident innate immune cells, such as Langerhans cells (LCs), mast cells (MCs), macrophages, γδ T lymphocytes, and innate lymphoid cells. Recent studies have demonstrated the importance of these immune cells in terms of producing different cytokines and other growth factors in corneal homeostasis and its pathologic conditions. This review paper briefly describes the latest information on these resident immune cells by specifically analyzing research from our laboratory.

## Introduction

Barrier surfaces, such as the skin and mucosal membranes, are major interfaces with the outside environment and play critical roles in immune surveillance (1, 2). At these sites, a cohesive network of individual tissues and cell types ensures barrier surface homeostasis and host protection from different environmental damages and infectious microbes (3–6). However, the different barrier surfaces have varying structural components due to different external challenges they face. In recent years, pioneer studies have revealed the unique immune characteristics of the different barrier sites (3, 5, 6).

Like other barrier surfaces, the cornea is a special surface between the inner eye tissue and the external environment (7). Its defense against microbes is mediated by two different types of immunity: innate and adaptive (8–11). First, histologically, the cornea comprises five different layers: the epithelium, Bowman’s membrane, the stroma, Descemet’s membrane, and the endothelium (12). The physical barrier formed by the tight conjunction between the corneal epithelial cells and the subtle structure of Bowman’s membrane prevents pathogens from further invading the corneal stroma (13). The expression of antimicrobial peptides from the epithelium also provides this defense (14). Moreover, the basal lamina of the cornea represents the final barrier against pathogen penetration (13). Second, a constant flow of tears and blinking can physically clean the corneal surface and wash away potential pathogens (15). Finally, the tear film contains diverse molecules with direct antibacterial activity, such as β-defensins and cytokeratin-derived antimicrobial peptides (14).

The immune cells controlling innate immunity mainly include dendritic cells (DCs), mast cells (MCs), macrophages, natural killer cells (NKs), γδ T cells, and innate lymphoid cells (ILCs). Corneal limbi have a rich distribution of capillaries and lymphatic vessels that serve as the entry and exit portals for immune cells (16, 17). Thus, the cornea is home to several immune cell populations that reside in both the central and peripheral corneal regions. These heterogeneous immune cell populations form a complex immune network that mainly comprises tissue-resident macrophages, Langerhans cells (LCs), MCs, lymphocytes, and ILCs. These immune cell subsets enable the cornea to respond to many environmental challenges by performing specialized functions (9). In recent years, our understanding of ocular surface immunology has been transformed by many new insights into both the ontogeny and function of most cornea-resident immune cells (7). Regarding the adaptive immunity and immunoregulation mechanism of the cornea, there are more detailed descriptions in other recent review papers (7–11, 18). Here, using our lab’s research work, this article only provides an overview of recent advances in understanding the diversity of the resident innate immune cell subsets of the cornea and discusses how these innate cells might be applied in corneal homeostasis and disease.

## LCs

LCs are an immune cell population located in different epithelial tissues (19). These cells form a network in the epithelium of the skin and mucous membranes (20). Because these cells reside in the corneal limbal and conjunctival epithelium in contact with the body and the environment, they were considered the first line cells of immune defense on the ocular surface (21). In recent years, our understanding of the origin, characteristics, and functions of LCs has changed considerably (22). LCs were once thought to be prototypes of DCs. Currently, LCs are thought to be a subgroup of macrophages that reside in the epidermis. To maintain their network, LCs, like macrophages in other tissues, are replenished by sustained low-level proliferation (about 5% of the population at a time) (23). However, unlike macrophages in other tissue species, LCs continue to migrate to the lymph nodes under homeostatic conditions to perform antigen presentation. The establishment and maintenance of the LC network depend on several important factors; for example, well-identified molecules include transforming growth factors (TGF)-b1 (24–26) and several transcription factors, such as *Runx3*, *ID2*, *PU.1*, and *P14* (27, 28). The development of LCs also depends on signals from IL-34, which are mediated by the colony stimulating factor (CSF)-1 receptor (29).

In development, LCs come from primitive macrophage progenitor cells during the embryonic period, mainly from the yolk sac and fetal liver (30). In the absence of inflammation, these cells maintain the stability of the cell population through *in situ* division (31). In addition, when the epidermis is subjected to severe disturbances and the LCs are damaged, the LCs may be replenished by monocytes from bone marrow sources (32, 33). These monocytes respond to an increase in the proinflammatory chemokine concentration in the epidermis. Interestingly, as the inflammation subsides, these monocyte-derived LCs are eventually displaced from the “historical stage” due to competition and are replaced by embryo-derived LCs.

**Figure 1:**
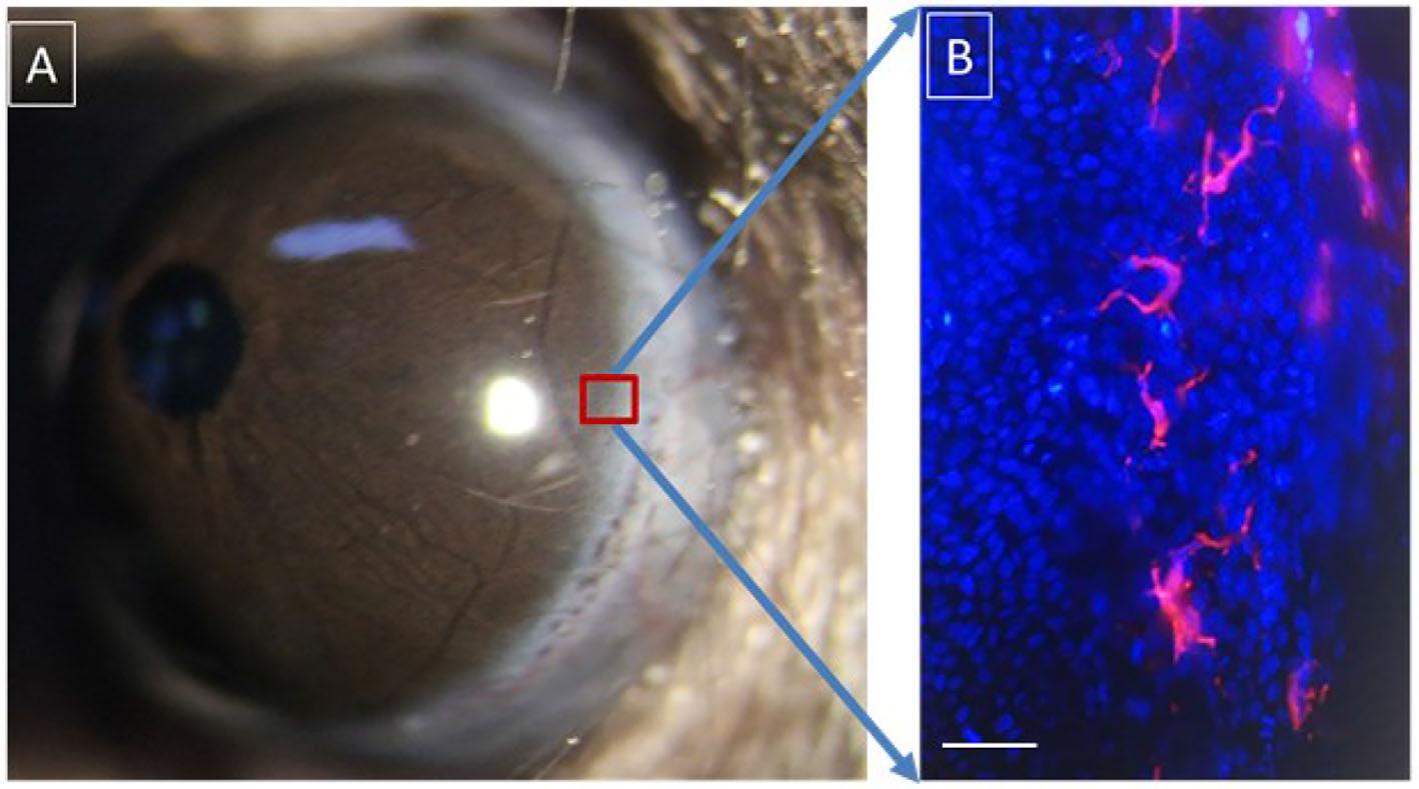
Langerhans cells located in the basal layer of the corneal limbal epithelium. **(A)** Anterior segment of murine eyeball; **(B)** Langerhans cells (phycoerythrin (PE)-conjugated anti-mouse CD11c staining, red) and basal epithelial cells (4’,6-diamidino-2-phenylindole (DAPI) staining, blue) in the murine corneal limbus. Scale bar: 25 μm.

Although LCs were discovered about 100 years ago, their exact role in immunology is controversial (34, 35). *In vivo* experiments have shown that LC protrusions are continuously stretched between epithelial cells repetitively, and their function may be to grab the surrounding antigens. In a resting state, only a few LCs continuously migrate to the lymph nodes of the drainage region after an antigen is seized. However, in an inflamed state, 10–20% of LCs migrate to the lymph nodes (36). Early studies suggested that LCs are a potential irritator of T-cells. This conclusion was drawn mainly on the analysis of *in vitro* experiments showing that the major histocompatibility antigen type II (MHC-II) expressed on LCs stimulates a mixed lymphocyte reaction in T-cells (37). A recent study of LCs showed that the C-type lectin receptor langerin (CD207) is a type of highly selective marker for LCs (38). After capturing an antigen as an antigen capture receptor, the langerin will internalize to form a LC-specific organelle called the Birbeck granule, believed to be a specialized antigen-processing compartment (38). This molecule can be specifically knocked out through genetic engineering techniques (39). However, the data obtained using this LC-deficient model provides inconsistent conclusions about the role of LCs in adaptive immune responses. The main conclusions from several studies are (40–42): (1) LCs migrate to the lymph nodes in the drainage region and trigger the tolerance of naive T-cells—even in an inflammatory context; and (2) for infection and antigen stimulation, the presence of LCs weakens the T-cell immune response rather than strengthening it. LCs play an immunosuppressive role in several immunologic disease models that have been proven, such as the contact hypersensitivity model (40, 41). Recently, it was reported that LCs produce regulatory T-cells (Tregs) when the skin is exposed to ionizing radiation (42, 43). However, in the muLangerin-diphtheria toxin receptor (DTR) model (langerin-positive cells express DTR and are depleted by intraperitoneally injecting diphtheria toxin), LCs did not show an immunosuppressive role, whereas they amplified the immune response to allergens (44, 45). Therefore, LCs have significant functional plasticity, and their response depends on the different immunological contexts (22, 46). LCs are widely found in the epithelium of the conjunctival and corneal limbus (21, 47, 48) (**Figure 1**). Although much research has been carried out on the role of LCs in many ocular surface diseases and some important conclusions have been drawn, restudies of mice with LC deficiency may yield more accurate and practical conclusions (49).

## MCs

In 1878, Paul Ehrlich first discovered MCs. It was not until the mid-twentieth century that researchers realized that MCs were involved in inflammation and allergic reactions (50, 51). However, the current evidence suggests that MC function is very broad and even closely related to non-immune diseases (52, 53). MCs are mainly distributed throughout the connective tissues of the body, especially in the barrier regions between the body and the external environment and also around the blood vessels, nerves, and lymphatic vessels (50). The development and survival of MCs require signaling from stem cell factors and the corresponding receptor c-Kit on the surface of the MCs (54). When the *c-Kit* gene is knocked out in mice, the development of MCs is greatly inhibited. Therefore, c-Kit-deficient mice are often used as animal models for MC deficiency (55). This deficiency can be partially reconstituted in function by intracutaneous, intraperitoneal, and intravenous transfer of adaptive MCs from normal animals (56). However, these c-Kit-deficient mice have also been associated with other defects because defects in the c-Kit signaling pathway also often cause abnormalities in the development of hematopoietic stem cells, thereby decreasing the number of red blood cells and neutrophils (57). To overcome this limitation, researchers developed models of MC deficiency that are independent of c-Kit (58, 59). The use of these models is likely to provide new information for clarifying the function of MCs.

Early studies demonstrated that MCs are closely related to allergic reactions (60). The surfaces of MCs have high-affinity immunoglobin (Ig) E receptors (61). When the allergen-specific IgE binds to these receptors, it degranulates the MCs. Preformed inflammatory molecules (such as histamines) within the cell are released to promote the inflammatory response by inducing vascular dilation and the vascular endothelial expression of adhesion molecules. Yet, recent studies have shown that MCs have diverse functions, including (62–65) (1) the promotion of the removal of pathogens by secreting antimicrobial peptides and activating phagocytes; (2) the degradation of endogenous toxic polypeptides and snake toxins; (3) the upregulation of immune regulation by releasing inflammatory cytokines to promote the migration, maturation, and differentiation of other immune cells; and (4) the downregulation of immune response *via* the production of IL-10.

**Figure 2:**
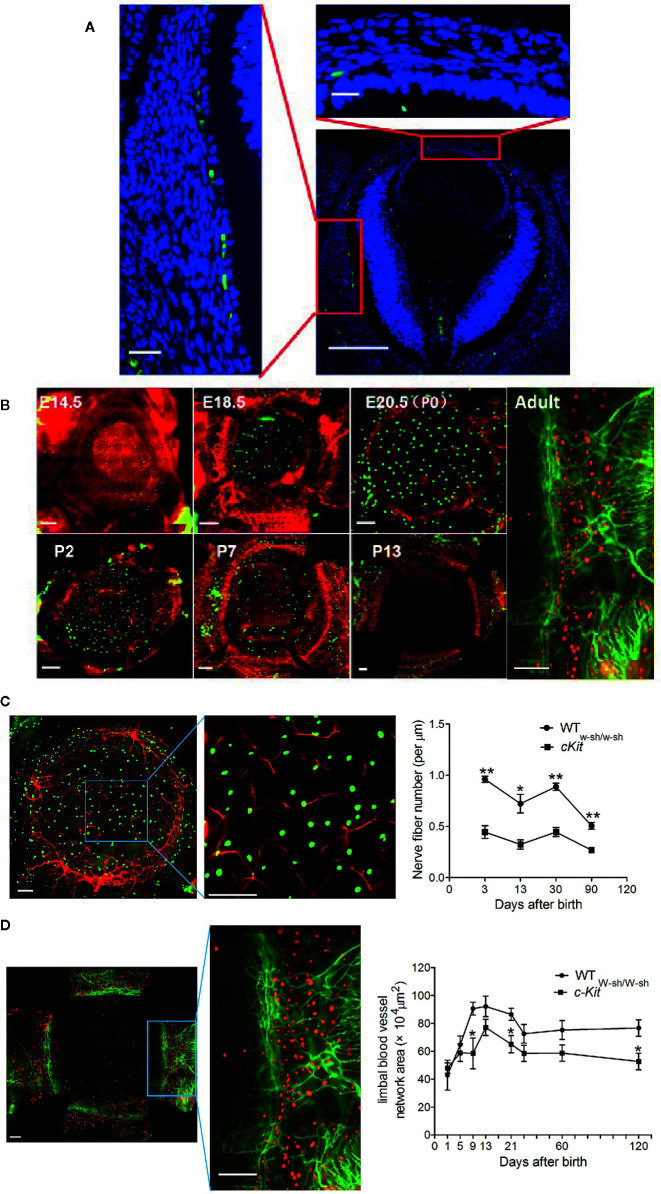
Dynamic changes in first-wave mast cells (MCs) during the embryonic and neonatal periods. **(A)** Immunostaining of eyeball cross section with fluorescein isothiocyanate (FITC)-conjugated avidin for mast cells and DAPI for cell nuclei. Scale bars: left and upper-right images, 20 μm; lower-right image, 100 μm. **(B)** These images depict immunostaining of the cornea with a complete limbus with anti-mouse CD31-PE (red) for blood vessels and FITC-conjugated avidin (green) for mast cells during different developmental stages from E14.5, P13, to Adult. Scale bars: 200 μm. E, embryonic day; P, postnatal day. [From Liu J et al. (67)]. **(C)** Analysis of the roles of MCs in corneal innervation. The left two images show the immunostaining of the cornea with NL557-conjugated anti-β-III tubulin and Avidin-FITC staining at P1. Scale bars: 200 μ m. The right image shows the differences in nerve fiber density between WT and c-Kit-/- murine corneas. **(D)** Analysis of the roles of MCs in limbal vasculogenesis. The left two images show the limbal vessel and MC immunostaining of the cornea with anti-mouse CD31-FITC and Avidin-PE staining, respectively. Scale bars: 200 μm. The right image shows the difference in limbal vessel network area between WT and c-Kit-/- murine corneas.

MCs were traditionally thought to stem from bone marrow (66). However, at the earlier stage of embryonic development, MC precursors are found in the fetal liver at E11. Consistent with this observation, we found that MCs exist in the presumptive cornea at E12.5 (**Figure 2A**) **(** 67), which is the time of definitive hematopoiesis in the fetal liver—not the bone marrow (68). Then, we confirmed two waves of MC emigration occurring at different developmental stages: the first wave occurs from at least E12.5 to postnatal day (P) 13 (eyelid opening time) (**Figures 2A, B** **)**, and the second wave occurs from birth to P13, stabilizing after P21 (67). The first wave has two stages: from E12.5 to birth and from birth to eyelid opening (P13) (67). MCs in the first stage were identified mostly in the presumptive corneal stroma (both central and peripheral), beginning as late as E12.5. The machineries underlying MC migration into the developing cornea are still unspecified. We also found that first-wave MCs in the first stage were proliferating (67). Thus, the steady increase of first-wave MCs during the first stage may be attributed to either the continuous trafficking of MC progenitors to the cornea, proliferation, or both (67). Interestingly, at day 13 after birth (time of eyelid opening), the MCs located in the central stroma of the cornea completely disappear within 24 h through an unknown mechanism (**Figure 2B**, P13) (67). Accordingly, eyelid opening motivates the disappearance of MCs in the cornea (67). However, the MCs located on the corneal limbus are long-standing and have a certain proliferation capacity. Importantly, compared with the classic functions of MCs involved in inflammation and allergic reactions, corneal MCs immigrated at the embryonic stage also exhibit special functions during corneal development. Our observations suggest that MCs may participate in corneal nerve growth by producing nerve-related growth factors such as neurotrophin (**Figure 2C**) and promote the growth of limbal blood vessels by producing vascular endothelial growth factor (**Figure 2D**) (67).

Conjunctival MCs are critical for the effector phase of allergic responses (69). Upon exposure to an allergen, allergen-specific IgE binds to the high-affinity receptor FceRI on MCs, triggering the degranulation of the proinflammatory mediators that cause increased vascular permeability and vasodilation (69). Limited studies have revealed that MCs are involved in the inflammatory reaction of the cornea induced by corneal injury (70). After corneal injury, the MCs located on the corneal limbus are activated and degranulated. This reaction is synchronized with the recruitment of neutrophils to the injured cornea. Further study showed that neutrophils are motivated by the attractive effect of CXCL2, a chemokine produced by MCs. Inhibiting the MC degranulation *via* the administration of cromolyn sodium will reduce the CXCL2 expression of MC and inhibit inflammation after corneal injury.

It has recently been found that MCs also play an important role in the course of corneal fungal infection (71). Remarkable MC degranulation in the limbus is present after corneal infection. The local administration of cromolyn sodium, an inhibitor of MC degranulation, significantly inhibits the dilation and permeability of blood vessels and prolongs the course of infection, as shown by much higher rates of corneal damage, fungus growth, and perforation (71). Interestingly, the inhibition of degranulation accompanies a decrease in the level of intercellular adhesion molecule-1 (ICAM-1) expression (71). ICAM-1 is one of the most crucial adhesion molecules required for the migration of inflammatory cells from the blood to the infected region. MCs are also involved in the immune rejection of corneal transplantation (72). The study by Li et al. found an increase in the number and activation of MCs on the corneal limbus after corneal transplant surgery (72). Pharmacologically inhibiting the activity of these cells with sodium cromoglicate can inhibit the migration of inflammatory cells to the transplant and the maturation of antigen-presenting cells trafficking to the transplant; reduce T helper (Th) 1 cytokine production and allosensitization in draining lymphoid tissues; decrease the graft infiltration of alloimmune effector cells; and extend the survival time of corneal transplants.

## Macrophages

Macrophages constitute a widely dispersed organ system in all vertebrate tissues (73), and they defend against microbes and remove dead and senescent cells acting as phagocytes. They also promote the homeostasis of different tissues *via* local trophic, regulatory, and repair functions. It has long been thought that macrophages in peripheral tissues come from the differentiated cells of circulating monocytes—that is, the continuous migration and replenishment of the monocytes from the bone marrow through the blood circulation (74). Recent studies integrating single-cell transcriptomics, genetic fate mapping, functional analyses, and imaging provide mechanistic evidence of prior observations showing that murine macrophage heterogeneity induces its diverse roles in tissue homeostasis and pathological response (75). Using genetic fate-map technology, macrophages in normal adult peripheral tissue were found to have at least three different macrophage subsets in their populations that had different origins (73, 76, 77). During organogenesis, macrophages derived from the yolk sac and fetal liver precursors were found to be throughout the tissue (78). These cells act as resident cells in adulthood and maintain the presence of this cell population by self-proliferation. Under normal steady-state conditions, these populations perform the localized clearance and nutritional functions of specific organs (79). However, some bone marrow-derived monocytes can be circulated into different tissues after birth to supplement these long-lived macrophage populations. When tissues are damaged and infected, more monocytes are recruited to the tissue and differentiated into macrophages (76–78).

The first subset of macrophages is produced during the primitive hematopoiesis of the yolk sac at E6.5–E8.5 (80). At E7.5–8.5, erythromyeloid progenitors (EMPs) are directly differentiated into macrophages and migrate into various peripheral tissues. These macrophages belong to primitive macrophages. After birth, this group of cells will maintain its presence mainly through local self-renewal. It has been determined that the microglia in brain tissue belong to this group. The second subset of macrophages develops during the fetal liver hematopoietic period (E11.5–15.5) as a part of the erythromyeloid progenitors that migrated from the yolk sac and began to express the transcription factor c-Myb (31). Under the control of c-Myb, this cell population is differentiated into embryonic monocytes, migrates to different tissues, and differentiates into macrophages. This group of cells also maintains self-stabilization *via* self-renewal after birth. The third group of macrophages appears during the bone marrow hematopoietic period (from E16.5 to birth). At E17.5 or after birth, hematopoietic stem cells (HSCs) are differentiated into monocytes, and some of these monocytes migrate into peripheral tissue and differentiate into macrophages (81, 82). These macrophages are bone marrow monocyte-derived macrophages. Thus, there are three different subsets of macrophages in different tissues. However, depending on the tissue environment, some subsets may exist alone, or all three subsets may coexist. For example, microglial cells in the brain tissue are the only source of macrophages from the embryonic yolk sac. In other tissues, such as liver, spleen, and lung tissues, there are combinations of three macrophage subsets of different origins at the same time.

**Figure 3:**
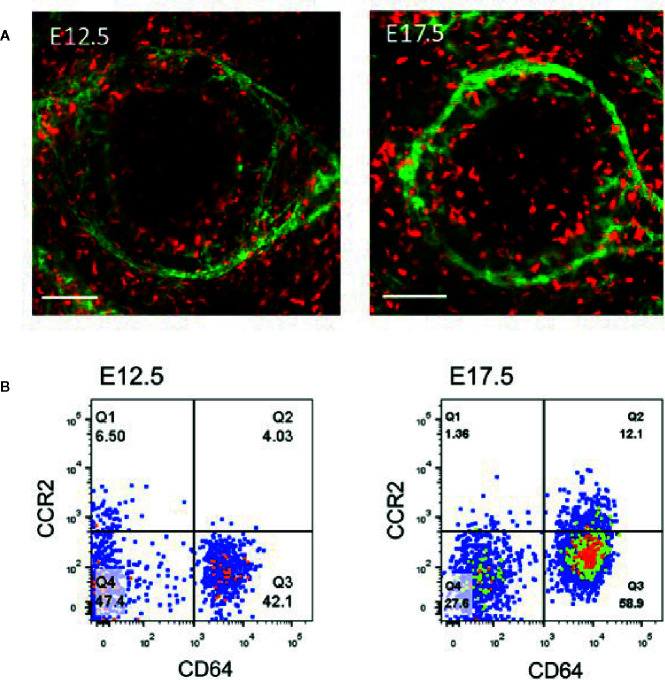
Analysis of corneal macrophages at E12.5 and E17.5. **(A)** Represented images of macrophage (PE-conjugated anti-mouse CD64^+^, red) distribution and limbal vessels (fluorescein isothiocyanate (FITC)-conjugated anti-mouse CD31, green) in E12.5 and E17.5 corneas. Scale bars: 200 μm [From Liu J et al. (86)]. **(B)** The cells in the flow dot plot of E12.5 and E17.5 were both derived from CD45 positive cells. CD64 antigen was used to identify macrophages, and CCR2 antigen was used to distinguished the distinct cell population in corneal macrophages.

Early studies showed that CD11b-positive macrophages in the cornea are the main resident immune cells in the cornea, accounting for 50% of all immune cells (83). These cells are mainly distributed throughout the cornea, including the center of the cornea and the corneal limbus (84). Through whole-mount immunostaining of the cornea and flow cytometric analysis, we identified the composition and distribution of macrophages in mice using the highly specific macrophage marker, CD64 (85), in the cornea (**Figure 3A**) and categorized these cells into C-C chemokine receptor (CCR) type 2^−^ and CCR2^+^ populations (86–89). Flow cytometric analysis of the corneal cells from the embryonic mice demonstrated that only CD64^+^CCR2^–^ macrophages were present in the corneas of the E12.5 mice, and CD64^+^CCR2^+^ macrophages were absent in the cornea until E17.5 (**Figure 3B**) **(** 86). Further study revealed that CD64^+^CCR2^−^ corneal macrophages were primarily maintained through local proliferation and were rarely replaced by donor blood monocytes (86). Conversely, CD64^+^CCR2^+^ corneal macrophages had a lower proliferation ability and were largely replaced by circulating monocytes (86). Therefore, unique maintenance mechanisms of different corneal macrophages exist depending on the tissue microenvironment and physiological context (86, 88, 89). Our recent study found that the distribution of the C64^+^CCR2^–^ macrophage population in murine cornea is influenced by gut microbiota (the microbe population living in the intestines) (88, 89). This alteration in the distribution of different macrophage subsets not only changes the development of the normal cornea after birth but also delays the regrowth of the corneal nerve fibers after corneal trauma (88, 89). Quantitative polymerase chain reaction (qPCR) analysis of flow cytometry-sorted corneal macrophages showed that CD64^+^CCR2^+^ corneal macrophages express representative genes (such as *IL-1β* and *TNF-α*) of M1-type macrophages (responsible for the initiation of inflammation) to promote the process of inflammation by secreting proinflammatory cytokines (86). However, CD64^+^CCR2^−^ corneal macrophages express representative genes (*IL-10*, *Arg1*, *Mrc1*, *Mgl1*, *Mgl2*, *Ym1*, and *Fizz1*) of M2-type macrophages (responsible for the inhibition of inflammation) (86). The depletion of CD64^+^CCR2^+^ corneal macrophages causes a decreased influx of neutrophils and the expression of inflammatory cytokines after corneal epithelial injury, whereas the depletion of CD64^+^CCR2^−^ corneal macrophages induces an increased neutrophil influx and the expression of inflammatory cytokines when compared with an undepleted control group (86). As expected, both treatments delayed corneal wound healing. Thus, these data indicated that CD64+CCR2+ corneal macrophages enhance the inflammatory response at the early stage of corneal wound healing, and CD64^+^CCR2^−^ corneal macrophages suppress the inflammatory response during the later stage (86). Interestingly, it was recently found that two different macrophage subsets in the cornea express different autonomic nerve receptors (87). While CD64^+^CCR2^–^ macrophages preferentially express the α-7 nicotinic acetylcholine receptor, CD64^+^CCR2^+^ macrophages preferentially express the β-2 adrenergic receptor (87). The topical administration of a β2AR agonist further enhanced the expression of the proinflammatory genes in the CD64^+^CCR2^+^ cell subset sorted from injured corneas. In contrast, the topical administration of an α7nAChR agonist further enhanced the expression of the anti-inflammatory genes in the CD64^+^CCR2^–^ subset (87). Thus, crosstalk between the autonomic nerve system and local macrophage populations is essential for the progress of corneal wound repair. Collectively, both macrophage populations play an important role in the appropriate repair of damaged corneal epithelium, and a deficiency in either one induces an imbalance in inflammation (86, 87).

Two recent studies from our lab further increased our understanding of two macrophage groups in the cornea (88, 89). Moreover, an increasing number of studies have shown that gut microbiota is closely linked with human health and disease (90)—not only by participating in the metabolism and absorption of nutrients (91) but also by tuning the development and response of the immune system (92). We induced gut dysbiosis with cocktail antibiotics after birth and found that normal gut microbiota was critical to the normal distribution of the CD64^+^CCR2^-^macrophage subset—rather than the CD64^+^CCR2^-^macrophage subset—in the cornea during development (89). Further studies have shown that the normal distribution of the CD64^+^CCR2^-^ macrophage subset during development significantly impacts morphological changes in the cornea, including corneal size, thickness, and nerve density. As predicted, improving or restoring gut microbiota—whether using probiotics or fecal transplants—can facilitate the recovery of various indicators of the corneal development process. Using a corneal wound model in adult mice, we found that antibiotic-induced gut dysbiosis can also inhibit every aspect of corneal wound repair, especially the recovery of corneal nerve density and sensory function, by reducing the number and distribution of CD64^+^CCR2^-^macrophage subsets in the cornea (88). Similarly, the use of probiotics and fecal transplantation to restore the gut microbiota composition can significantly improve the above inhibited repair process. In summary, these data further highlight the critical importance of the CD64^+^CCR2^-^macrophage subset for maintaining normal corneal integrity post-wound repair.

## γδ T-Cells

Lymphocytes, also called T-cells, not only continuously circulate between the blood and lymphoid organs but also settle in nonlymphoid tissues. These resident lymphocytes are prominently distributed at barrier sites, including the mucosal surfaces and the skin (93, 94). Most of these lymphocytes are unconventional T-cells, such as γδ T-cells, innate lymphoid cells, and tissue-resident memory T-cells. Important properties shared by tissue-resident lymphocytes include the following (4, 95, 96): (1) long-term maintenance and self-renewal; (2) high abundance in barrier tissues; (3) ability to sense microbial products, cytokines, alarmins, and stress ligands; and (4) the rapid provision of antimicrobial and tissue-protective factors. Although these different cell types differ in biology, they still share common functions, including maintaining tissue integrity and fighting damage caused by infection and noninfectious stimulation.

γδ T-cells represent a prominent, innate T-cell subset expressing the γδ T-cell receptor (TCR) (96). In both humans and mice, γδ T-cells are generated in the thymus from CD4^−^CD8^−^ double-negative (DN) progenitor cells (97). These DN cells commit to the αβ or γδ T-cell lineage depending on the type of V(D)J rearrangements and the strength of the pre-TCR signal (98, 99). In mice, the recombination of specific Vγ and Vδ segments in the TCR is performed in a highly orderly manner during embryonic development (100, 101). This leads to the emergence of γδ T-cells with oligoclonal or monoclonal TDRs, which reside in different epithelial tissues. However, new experimental evidence has challenged this concept. This study shows that, in the early stages of embryo development, γδ T-cells in the epidermal tissue originate from yolk sac hematopoiesis to settle in different epidermal tissues (102). Unlike the αβ TCR, the γδ TCR has a longer immunoglobulin-like complementarity determining the region 3 (CDR3) loop. Also, in contrast to antigen recognition by αβ T-cells, γδ T-cells do not have strict MHC restrictions for antigen recognition (103). Thus, these heterogeneous γδ T-cell subsets identify ligands as diverse as lipids represented by MHC class I-like molecule CD1 family members (CD1a, CD1b, and CD1c). Thus far, the characteristics of the ligands recognized by γδ TCR are yet unclear (96).

Most γδ T-cells reside in barrier tissues, such as the mucosal membranes, and provide a first-line immune defense to external stress events (4). γδ T-cells are also multifunctional immune cells that play an important role in tumor immune surveillance, wound repair, and autoimmunity (96). Some researchers divide γδ T-cells into early-occurring, natural-type γδ T-cells (natural γδ T-cells) and induced γδ T-cells (inducible γδ T-cells) that appear during the postinfection period depending on the time of occurrence of the anti-infection response (104).

**Figure 4:**
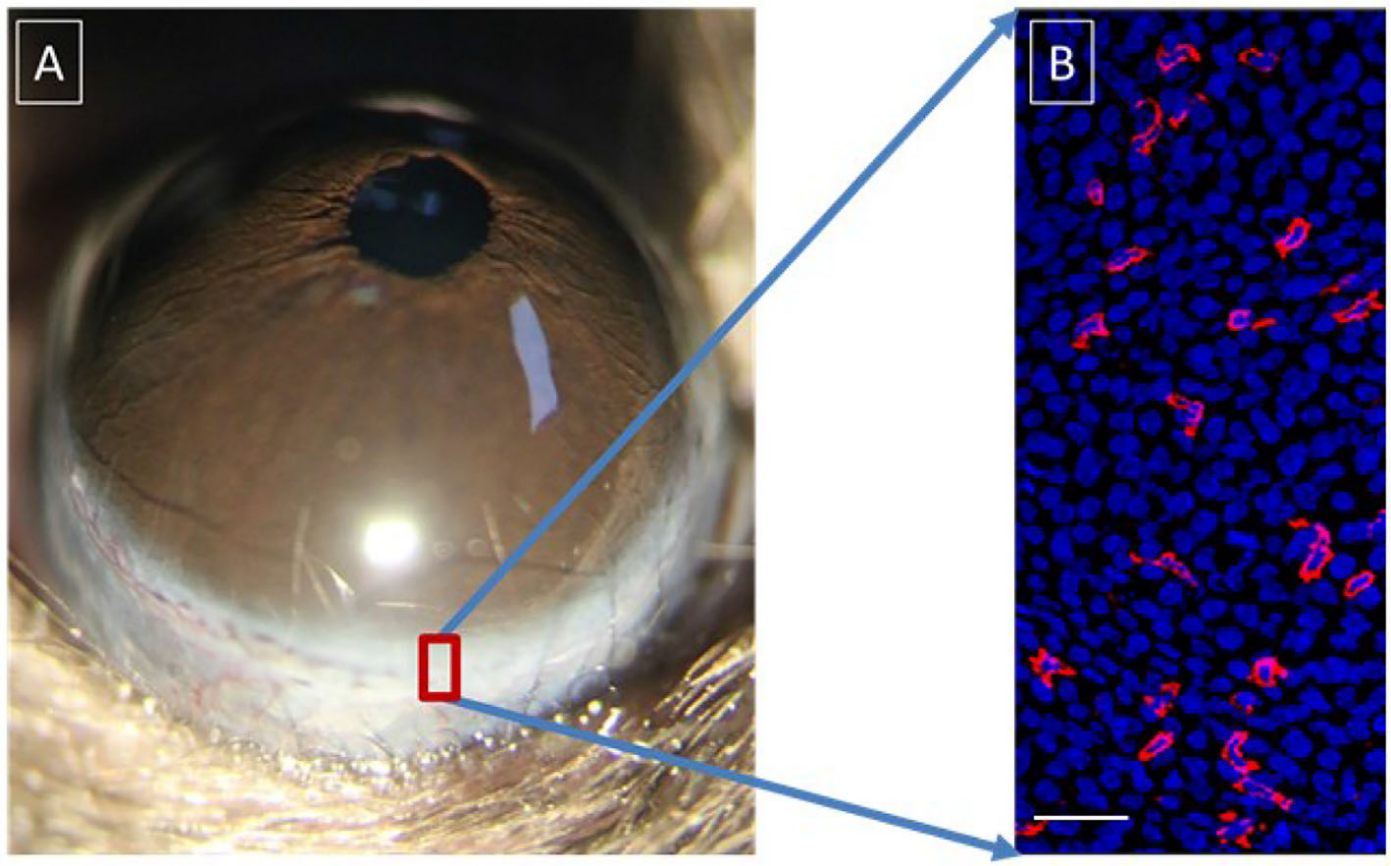
Distribution of γδ T-cells in the corneal limbus and conjunctiva. **(A)** Anterior segment of murine eyeball; **(B)** γδ T-cells (PE-conjugated anti-mouse TCRγδ, Clone GL3, red) and epithelial cells (DAPI staining, blue) in the murine corneal limbus. Scale bar: 25 μm.

γδ T-cells are widely present in many epithelial tissues, such as the skin and various mucous membrane epithelia. Recent studies from our laboratory and others have shown the presence of γδ T-cells in the epithelium of the ocular surface (**Figure 4**) (105–108). These T-cells play a vital role in maintaining the stability of ocular surface homeostasis and the corneal wound repair process (106). There may be two main mechanisms behind their functions: (1) the production of cytokines, such as fibroblast growth factor 7 (FGF7), FGF9, and insulin-like growth factor, which act directly on epithelial cells through specific receptors (109) and (2) the production of interleukin (IL)-17A and IL-22. IL-17A is released by γδ T-cells after corneal wounding and contributes to inflammation by enhancing neutrophil infiltration in the injured cornea and the production of proinflammatory chemokines (105). Corneal epithelial cells express high levels of IL-22 receptors (106). Therefore, when these γδ T-cells become activated, the secreted IL-22 directly stimulates the division of the corneal epithelial cells and produces substances, such as antimicrobial peptides, to protect the integrity of the ocular surface. There is evidence that these cells are partly responsible for the pathogenesis of the ocular allergic inflammatory response (110), corneal transplantation (111), and fungal keratitis (112).

## Innate Lymphoid Cells

One breakthrough in immunology over the past decade was the discovery of ILCs (113). These cells comprise three or more heterogeneous lymphoid cell subsets, lack T- and B-cell antigen-specific receptors, and respond quickly to invasive pathogens and wounding. These cells are derived from a common lymphoid progenitor that lacks the expression of lineage (Lineage, Lin) marker molecules (T-cell receptors, B-cell receptors, myeloid and/or DC markers). Recently, an analysis of the developmental stages of the ILC population showed that the ILCs basically included two large groups of cytotoxic NKs and noncytotoxic ILCs (114). The former—mainly through a perforin mechanism that kills infected cells—participates in antiviral infection. The latter is further divided into three subgroups—type I ILCs (ILC1s), type 2 ILCs (ILC2s), and type 3 ILCs (ILC3s)—according to the different necessity for transcription factors during development (115).

NK cells and ILC1s have different functions (116). NK cells are cytotoxic cells and kill virus-infected normal and tumor cells, and they mirror the functions of CD8^+^ cytotoxic T cells (117). However, ILC1s are noncytotoxic and function as a first line of defense against infections caused by viruses and certain bacteria (118). ILC1s are resident cells, whereas NK cells circulate in the bloodstream. The development of ILC1s strictly depends on the transcription factor T-bet, whereas NK cells can develop in T-bet-deficient hosts (119). In addition, NK cells require the T-box factor Eomes, whereas ILC1s can develop without this transcription factor. Thus, Eomes expression is often used as a marker for NK cells. ILC1s produce IFN and TNF and are involved in responses to intracellular bacteria and parasites. IFN-γ released by NK cells stimulates the expression of Th1 chemokines (CXCL9, CXCL10, and CXCL11) in the corneal and conjunctival epithelium in response to experimental desiccation (120). Cytokines IL-12, IL-15, and IL-18 trigger their activation. ILC1s mirror Th1 in adaptive immunity. The development of ILC2s depends on the transcription factor GATA-3. IL-33, TSLP, and IL-25 trigger ILC2 activation *via* the NF-κB and MAPK pathways. After activation, ILC2s produce Th2 cytokines IL-4, IL-5, IL-9, and IL-13 (121). More importantly, ILC2s also produce amphiregulin (AREG), a member of the epidermal growth factor family, which is critical in epithelial wound healing (64). Conversely, some cytokines, such as type 1 IFNs, IFN-γ, and IL-27, inhibit the activation of ILC2s *via* the STAT1 signaling pathway (122). ILC2s mirror Th2 cells in adaptive immunity. ILC2s are mainly involved in wound repair, allergic inflammation, parasite infection, and metabolic homeostasis (123). The development of ILC3s depends on the transcription factor RORγt. IL-1β and IL-23 induce ILC3s to become activated to produce IL-17A, IL-22, or both. ILC3s mirror Th17 and Th22 cells in adaptive immunity. They mainly participate in the immune response to extracellular bacteria, wound repair, and the development of lymphatic tissue. Also, ILC3s can regulate adaptive Th17 cell responses.

**Figure 5:**
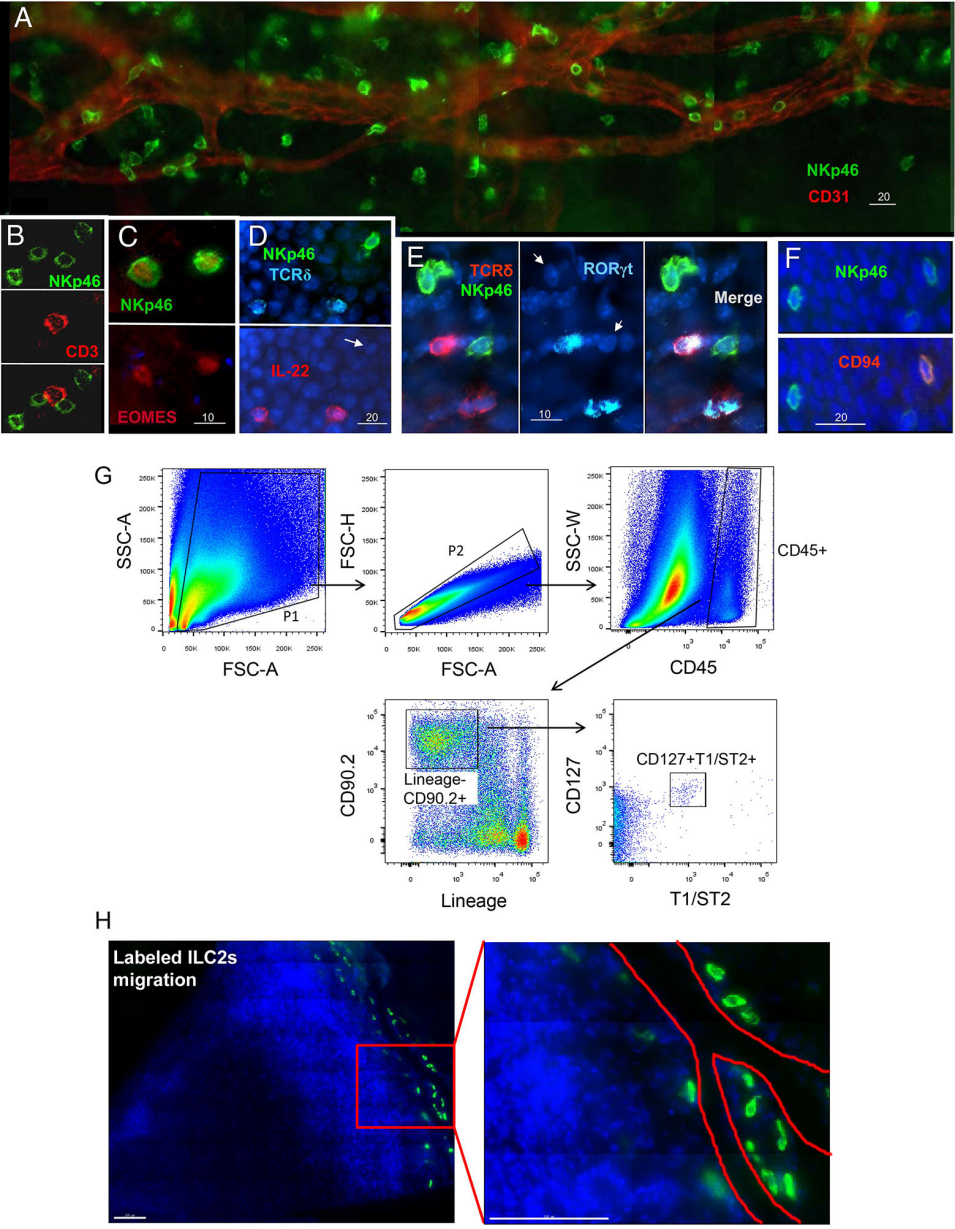
Identification of NK cells and ILC2s in the cornea. **(A–F)** Immunostaining and phenotypical identification of corneal limbal NK cells with anti-mouse NKp46-FITC, CD3-PE, EOMES-PE, IL-22-PE, RORγt-APC, and CD94-PE in the injured cornea at 24 h after corneal abrasion. (From Liu Q et al. (125). **(G)** The lineage antibody is a cocktail of anti-mouse CD3, anti-mouse Ly-6G/Ly-6C, anti-mouse CD11b, anti-mouse CD45R/B220, and anti-mouse TER-119 antibodies. ILC2s were identified as a CD45^+^Lin-CD90.2^+^CD127^+^T1/ST2^+^ cell population. **(H)** After corneal epithelial wounding, anti-CD90.2 antibody–treated mice were injected i.v. with sorted lung ILC2s stained by carboxyfluorescein diacetate succinimidyl ester. Corneas of those mice stained with DAPI were visualized using fluorescence microscopy 24 h after injection. The red lines represent the limbal vessel wall. Scale bars: 100 μm. (From Liu J et al. (126).

ILCs are tissue-resident cells and are integrated into the fabric of tissues. Although the characteristics of ILCs in many tissues have been identified, there has been little research on their presence and characteristics in ocular tissue. Several recent studies, including those from our laboratories, have confirmed NK cell subsets in both the normal conjunctival (124) and corneal limbi (125). These NK cell subsets are phenotypically NKp46^+^, NK1.1^+^, NKG2D^+^, EOMES^+^, CD3^–^, CD94^–^, RORγt^–^, IL-22^–^, and CD127^–^, consistent with a subset of classic NK cells (**Figures 5A–F**) **(** 125). These NK cells might have multiple effects on the ocular surface. First, they produce Th17 and INF-γ to participate in the occurrence and development of dry-eye disease (108) and the neovascularization of the ocular surface (127). Second, we found that depleting NK cells or blocking NKG2D receptors significantly increases the accumulation of neutrophils in the wounded cornea and delays the reepithelialization and regrowth of the corneal nerves following a corneal abrasion. However, the depletion of neutrophils will not reduce NK cell accumulation in the injured cornea. Thus, our data support a new concept that NK cells indirectly support corneal healing by preventing the excessive recruitment and tissue damage of neutrophils.

The role and distribution of other ILC subtypes on the normal ocular surface are yet unclear. Recently, our data from a mouse model showed that the ocular surface tissue at least has ILC2s (126). Further study found that this cell population phenotypically expresses CD127, T1/ST2, and CD90 and is relatively rare in resting corneas (**Figure 5G**). However, the population increases in number following a corneal epithelial abrasion. These cells are mainly distributed around the corneal blood vessels (**Figure 5H**). Depletion of this cell population through antibodies causes delayed corneal wound repair, whereas the local adoptive transfer of ILC2s partially restores the healing process. Further analysis reveals that IL-25, IL-33, and thymic stromal lymphopoietin play a critical role in corneal ILC2 responses following corneal injury and that CD64^+^CCR2^–^ corneal macrophages are essential producers of IL-33 in the cornea (126). Collectively, these data reveal the essential role of cornea-resident ILC2s in the recovery of corneal epithelial integrity following an acute injury (126). However, the role of the other two cell populations, ILC1s and ILC3s, in the onset of other ocular surface diseases needs to be further explored.

Finally, our understanding of ILC function to date has relied primarily on immunodeficient mice (such as Rag-defective mice) or on models that use non-ILC-specific antibodies to remove an ILC subset. Therefore, future studies should build new models of ILC-subset deficiency to illustrate the contribution of individual cell populations to skin and mucous membrane defense. Importantly, a greater number of ILC subgroups may be found in the future. For example, a regulatory subpopulation of ILCs, called ILCregs, was recently identified in the intestine; it suppresses the activation of ILC1s and ILC3s through the production of IL-10 to avoid innate intestinal inflammation (128).

## Conclusions

The cornea is rich in innate immune cells, making it a special surface of the body. These immune cells, together with corneal cells, form a complicated network to protect against damage from hostile environments and the invasion of pathogenic microorganisms. In recent years, our understanding of the types of immune cells in the cornea has increased rapidly. LCs, MCs, macrophages, γδ T-cells, and ILCs play different roles in corneal homeostasis, wound repair, pathogen detection, and cornea response. Newly discovered cells, such as ILCs, and the re-evaluation of classic immune cell functions in various corneal diseases will provide insight into the exploration of new targets and measures for treating many ocular surface diseases.

## Author Contributions

ZL conceptualized, wrote, reviewed, and revised this manuscript. JL wrote this manuscript, prepared images, and formatted the text and references. All authors contributed to the article and approved the submitted version.

## Funding

Research support was provided by the National Natural Science Foundation of China through Grants 81470603 (to ZL) and 81770962 (to ZL); the Ministry of Science and Technology of the People’s Republic of China through Grant 2018YFC0114500 (to ZL), a PhD Start-Up Fund of the Natural Science Foundation of Guangdong Province of China through Grant 2018A030310605 (to JL), and the China Postdoctoral Science Foundation through Grant 2017M622913 (to JL).

## Conflict of Interest

The authors declare that the research was conducted in the absence of any commercial or financial relationships that could be construed as a potential conflict of interest.
